# Overview of systematic reviews of health interventions that aim to prevent and treat overweight and obesity among children

**DOI:** 10.1186/s13643-022-02047-7

**Published:** 2022-08-13

**Authors:** Edgar Denova-Gutiérrez, Lucía Méndez-Sánchez, Berenice Araiza-Nava, Alejandra González-Rocha, Teresa Shamah, Anabelle Bonvechio, Simón Barquera, Juan Rivera

**Affiliations:** 1 Nutrition and Health Research Center, National Institute of Public Health, Cuernavaca, Mexico; 2 Clinical Epidemiology Research Unit, Hospital Infantil de Mexico Federico Gomez, Faculty of Medicine of National Autonomous University of Mexico (Universidad Nacional Autónoma de México), Mexico City, Mexico; 3 Center for Research in Evaluation and Surveys, National Institute of Public Health, Cuernavaca, Mexico

**Keywords:** Obesity, Overview, Systematic review, Overweight, Prevention, Health interventions, Children

## Abstract

**Background:**

Childhood overweight and obesity is a global public health issue. Although there is evidence of a reduced prevalence in some countries, there is still much controversy about the efficacy of health interventions that aim to prevent and treat obesity in this specific population. The objective of the present study is to develop an overview of systematic reviews (OSRs) that assesses the effects of school-based, family, and multi-component health interventions for the prevention and treatment of obesity, change in physical activity, dietary, and/or hydration behaviors, and change in metabolic risk factors in school-aged children.

**Methods:**

This protocol was developed using the methodology proposed by Cochrane. It outlines a comprehensive search in 12 electronic databases to identify systematic reviews of health interventions, including studies that evaluate and how to prevent and/or treat overweight and/or obesity in children aged 6 to 12 years. The risk of bias of the included Systematic Reviews will be assessed with the ROBIS tool.

**Discussion:**

Since the OSRs methodology’s purpose is only to harmonize evidence from open access publications, ethical consent is not necessary for the present protocol. In terms of diffusion, a paper will be submitted for publication in a scientific journal to describe the main results obtained through the OSRs.

**Trial registration:**

The present overview of the systematic review protocol has been registered in PROSPERO (ID number 218296).

## Background

Childhood obesity is a global public health problem. Since 1975, epidemiological statistics indicate that this problem has nearly tripled worldwide [1]. In 2016, over 340 million children and adolescents (5–19 years) were overweight or obese, and this disease has risen dramatically from 4 to 18% in the last three decades [1]. The rate of increase childhood obesity in many countries is alarmingly greater than the rate in adults, even though the prevalence of obesity in this population is projected to be lower than the adult prevalence [2]. These trends are expected to continue if no radical actions to tackle the epidemic are implemented.

Obesity in childhood can pose an imminent threat to children’s health as it has been associated with multiple metabolic conditions (i.e., hyperlipidemia, hypertension, and abnormal glucose tolerance, as well as other illnesses) [3, 4]. In addition, psychological issues (i.e. attention deficit, impaired educational attainment, and hyperactivity disorder) and social stigmatization can affect children who live with obesity, impacting their physical and mental health [5]. It can also have substantial long-term health consequences into adulthood. Hence, the risk of having obesity as an adult is double for children with obesity compared to those who do not have this condition. Furthermore, they have a greater risk of experiencing more severe health outcomes, such as hypertension, type 2 diabetes, and cardiovascular diseases, among others [6, 7].

Schools are conducive settings for the promotion of healthy behavior; thus, there great interest in school-based interventions for the prevention of childhood obesity, and the proof is the high volume of related evidence produced in the last years. Bahia et al. 2019 [8] recognized this high volume of research in the field as an obstacle for decision-makers, so they conducted an overview of systematic reviews (OSR) to try and clarify the available evidence through a meta-analysis of health interventions in children and adolescents. They found six systematic reviews (SRs) of interventions aimed at prevention, 17 for treatment, and one with mixed interventions (prevention and treatment); only four of these were considered to be of high quality. The outcomes assessed were weight, body mass index *Z*-score (BMI *Z*-score), fat content, fat distribution, anthropometric measures, dietary behavior, sedentary behavior, physical activity behavior, and cardiovascular risk factors. Prevention interventions did not show any significant effect on BMI *Z*-score when compared with control, but nutritional education, TV screen time, and physical activity did produce modest short-term weight reduction. Treatment interventions such as diet, physical activity promotion, supervised exercise, lifestyle, or multi-component interventions (including different interventions such as diet, physical activity, education, the use of digital technologies for public health “m-health”, in different settings such as school, family or the community), and school-based education in ten SRs were associated with a reduction in the main outcomes. Mixed interventions for treatment showed improvements in BMI *Z*-score, but these were not consistent across the reviews. These OSRs describe great heterogeneity and low quality of primary studies reported by the SRs authors, stating the need to combine different approaches across settings to effectively control the obesity epidemic. Amini et al. 2015 [9] also tried to summarize the existing SRs evidence on the effect of school-based interventions to control childhood obesity. They concluded that multi-component interventions appear to be superior to single ones in terms of adiposity reduction, but differences must be considered by sex, psychological, physiological, and cultural aspects. Also, these OSRs report that sustainability and evaluation of adverse or unwanted effects are essential in the study of effectiveness. Bussiek et al. 2018 [10] also developed an OSRs protocol to summarize the existing evidence in child and adolescent prevention interventions, with changes in behaviors and the BMI *Z*-score as the main outcomes. One of the issues surrounding OSRs is that the methodology for its development is currently vague, even though there have been efforts to clarify it [11–15]. Ells et al. presented an OSRs for children and adolescents, part of their findings is the vast majority presented lifestyle interventions (141/163 trials), showing us the opportunity to look deeper into those kinds of interventions [16]. Also, recently a position paper based on an umbrella review, about the interventions and prevention of pediatric obesity and overweight, suggest prevention from the school interventions and regulated screen time for children (6–12 years) but it is necessary deep information about the interventions and focus on this age group [17, 18].

OSRs would allow us to provide a general view of a public health problem that is widely studied by the scientific community and facilitate the decision-making process, also guiding future research. Conducting an OSRs with a focus on a specific group of age gives the opportunity to deepen the interventions of prevention and treatment. Therefore, considering the advances that have been made in researching this topic, it is necessary to develop an OSR that identifies effective public health strategies to prevent and manage childhood obesity. It must also communicate the impact of unique and multi-component lifestyle interventions that consider socio-cultural and economic context, as well as the quality of the existing evidence. This could provide a comprehensive and non-fragmented view of the problem that enables researchers and policymakers to generate new, feasible approaches to address obesity in this population.

Thus, the present protocol for an Overview of Systematic Reviews provides a methodology to summarize systematic reviews that assess the effects of school-based, family, and multi-component health interventions to prevent and treat overweight and obesity among school-aged children.

## Methods

This Overview of Systematic Reviews follows the methodology proposed by The Cochrane Collaboration [14]. Additionally, registration is in PROSPERO (ID number 218296). Two reviewers will be involved in pilot testing for the search strategy (DG-E, MS-L), independent screening by duplicate (MS-L, AN-B), selection, overlapping, and extraction process, also by duplicate (GR-A, AN-B).

### Inclusion criteria for systematic reviews

SRs of interventions will be included. Studies evaluating health interventions to prevent and/or treat overweight and/or obesity in children aged 6 to 12 years will be considered. These interventions may be based in school, family, and primary care settings (including mixed settings). SRs that included controlled trials, non-randomized trials (aiming to detect health interventions in this age group), or interventions that evaluate public health policies applied in this population will be analyzed, and those clinical trials will be presented separately. To be considered, SRs must report at least one of the following outcomes: (a) a change in weight, (b) a change in BMI Z-score, (c) a change in body composition and anthropometric measures (i.e., body fat percent, waist circumference), (d) change in metabolic risk factors (i.e., blood pressure, total cholesterol, high-density lipoprotein cholesterol, low-density lipoprotein cholesterol, triglycerides, and/or glucose), (e) change in physical activity, dietary and/or hydration behaviors, (f) psycho-social changes. For a study to be considered an SR it has to have: (1) perform a comprehensive literature search in at least three electronic databases; (2) utilize paired independent reviewers in multiple stages; (3) undergoes a critical assessment; and (4) complete a risk of bias assessment. Studies that do not comply with these characteristics will not be considered an SR. In addition, studies that do not include a stratified analysis of children from 6 to 12 years of age, that carry out pharmacological or surgical interventions will be excluded from this overview.

### Search methods to identify reviews

Previously validated search strategies, which are shown in Table 1, will be used to perform the search for SRs in electronic databases, also, different terms will be used as adapted per database for example; (obesity) AND (overweight) AND (prevention) AND (treatment) AND (child) for PubMed. The electronic search will be conducted in the following databases; PubMed, Embase, The Cochrane Library, LILACs, CINAHL, PsycINFO, PROSPERO, OT Seeker, TripDatabase, DARE, Epistemonikos, and Health Interventions. Additionally, a manual search will be conducted for scanning references lists of the preview overviews, similar overviews and the included SRs. The search will be performed with no language restriction up until December 2021. The descriptors included will be obesity, overweight, treatment, and prevention. The results of these searches will be assessed by title and abstract by two independent reviewers [MS-L, AN-B], and all the relevant citations will be retrieved for full-text review. The same two independent reviewers will assess the full-text articles for potential inclusion. In the event of disagreement, a third author will decide whether or not to include the article (DG-E).

**Table 1:** Search strategies

PubMed (MEDLINE)	((((obesity) AND (overweight)) AND (prevention)) AND (treatment)) AND (child) Filters: Systematic Review Sort by: Most Recent
EMBASE	Systematic review AND child AND obese and overweight AND prevention AND treatment - filter: Reviews
LILACS	obesity AND overweight AND prevention AND treatment AND child - Tipo de estudio: Rev Sistemática
CINAHL	obesity AND overweight AND treatment AND prevention AND ( (TI (systematic* n3 review*)) or (AB (systematic* n3 review*)) or (TI (systematic* n3 bibliographic*)) or (AB (systematic* n3 bibliographic*)) or (TI (systematic* n3 literature)) or (AB (systematic* n3 literature)) or (TI (comprehensive* n3 literature)) or (AB (comprehensive* n3 literature)) or (TI (comprehensive* n3 bibliographic*)) or (AB (comprehensive* n3 bibliographic*)) or (TI (integrative n3 review)) or (AB (integrative n3 review)) or (JN “Cochrane Database of Systematic Reviews”) or (TI (information n2 synthesis)) or (TI (data n2 synthesis)) or (AB (information n2 synthesis)) or (AB (data n2 synthesis)) or (TI (data n2 extract*)) or (AB (data n2 extract*)) or (TI (medline or pubmed or psyclit or cinahl or (psycinfo not “psycinfo database”) or “web of science” or scopus or embase)) or (AB (medline or pubmed or psyclit or cinahl or (psycinfo not “psycinfo database”) or “web of science” or scopus or embase)) or (MH “Systematic Review”) or (MH “Meta Analysis”) or (TI (meta-analy* or metaanaly*)) or (AB (meta-analy* or metaanaly*)) ). Narrow by SubjectAge: - all child
PsychINFO	(obesity and overweight and prevention and obesity and child and systematic review).mp. [mp=title, abstract, heading word, table of contents, key concepts, original title, tests & measures, meshlimit 2 to ("reviews (best balance of sensitivity and specificity)" and 180 school age <age 6 to 12 yrs> and "0110 peer-reviewed journal")
PROSPERO	child and obesity or overweight and treatment or prevention
TripDataBase	(title:child)(title:obesity OR overweight)(title:prevention OR treatment)
DARE	(child) AND (obesity OR overweight) AND (prevention OR treatment) IN DARE
OTSeeker	[Any Field] like 'child' AND [Any Field] like 'obesity' OR [Any Field] like 'overweight'
Epistemonikos	"(title:((title:(child) OR abstract:(child)) AND (title:(obesity) OR abstract:(obesity)) OR (title:(overweight) OR abstract:(overweight)) AND (title:(treatment) OR abstract:(treatment)) OR (title:(prevention) OR abstract:(prevention))) OR abstract:((title:(child) OR abstract:(child)) AND (title:(obesity) OR abstract:(obesity)) OR (title:(overweight) OR abstract:(overweight)) AND (title:(treatment) OR abstract:(treatment)) OR (title:(prevention) OR abstract:(prevention)))) (title:((title:(child) OR abstract:(child)) AND (title:(obesity) OR abstract:(obesity)) OR (title:(overweight) OR abstract:(overweight)) AND (title:(treatment) OR abstract:(treatment)) OR (title:(prevention) OR abstract:(prevention))) OR abstract:((title:(child) OR abstract:(child)) AND (title:(obesity) OR abstract:(obesity)) OR (title:(overweight) OR abstract:(overweight)) AND (title:(treatment) OR abstract:(treatment)) OR (title:(prevention) OR abstract:(prevention)))) Publication type: systematic reviews"
Health Evidence	"[obesity AND overweight AND prevention AND treatment] AND Limit: Population = Grade school aged (5-12 years) Topic Area = Chronic Diseases -> Obesity, Health Through the Ages -> Youth Health, Nutrition, Physical Activity, Social Determinants of Health (e.g., social environments, education, employment and working conditions)"
The Cochrane Library	(obesity):ti,ab,kw AND (overweight):ti,ab,kw AND (prevention):ti,ab,kw AND (treatment):ti,ab,kw AND ("Child"):ti,ab,kw

### Data collection and analysis

#### Selection of reviews

All relevant Cochrane and non-Cochrane SRs that match the previously mentioned criteria will be selected. The SRs selected for inclusion will be assessed to identify duplicate studies using a reference matrix (overlapping process) and the corrected area will be calculated following the proposed methods by Pieper et al. 2014 [15]. This method ensures that no outcome data is double-counted and that all outcome data from relevant SRs are included. The selection of the studies will be realized by an excel spreadsheet designed by the researchers that collaborate on this overview. As noted previously, a pilot testing was conducted and consensus meetings for disagreements.

Retrieved protocols will be checked for publication status and in specific cases, authors will be contacted to confirm the progress or publication status. When the scope of the included SRs is wider than ours, subsets of information regarding our target population (school-age children from 6–12 years old) or outcomes will be retrieved.

#### Data extraction and management

Data extraction will be performed independently by the two reviewers in a predefined platform, retrieving the following information: Author, year, the language of publication, date last assessed as up-to-date, objective, number of included studies, author’s information of the included primary studies, country of publication, the studied population, types of studies included, SR search strategies, names of databases searched in each SRs; date ranges of databases searched in each SRs; date of last search update in each SRs, participant characteristics such as age, sex, ethnicity, stage of the disease, co-morbidities; definition of disorder; type of intervention (s), time of application, frequency, intensity and dose, the follow-up time, setting, target population of the intervention (s), primary and secondary outcome (s), adverse events, the risk of bias of the included primary studies, quantitative outcomes data, the certainty of the evidence, limitations, conflicts of interest, and funding source.

Data analysis will be stratified by the objective of the health intervention, being either prevention and/or treatment. Subgroup analysis will be performed by the type of outcome measure and type of setting where the health intervention(s) is done. As the main goal is to present and describe the body of evidence currently available, all outcome data will be presented as extracted from the SRs, and no re-analysis will be performed.

Narrative summaries will be presented by the objective of the intervention (treatment or prevention) per SR, also, as summary tables of the findings of each SR. The health interventions will be categorized by their effectiveness or clinical importance as far as possible; with a summary table of findings as a preliminary synthesis of the included studies and identifying: (1) how the intervention works, (2) why, and (3) for who. To assess and report the certainty of the evidence found, the GRADE [19] assessments presented in the included systematic review will be extracted and reported. If the information is not available, it will be reported as not available data.

### Assessment of methodological quality of included reviews

The assessment of the methodological quality of the included reviews will be performed independently by two reviewers using the ROBIS tool [20] and a summary developed with the same visualization tool. The three phases contemplated in the ROBIS tool will be assessed for each included SR using pre-formatted extraction forms, which will be presented in tables consensus meetings will be conducted between the researchers for discordant appraisals. Also, data on the risk of bias of each primary study contained in the included SRs will be extracted and presented as a summary by domain. Considering the possibility of having different instruments used in the primary studies, the results will be presented and summarized in a narrative and tabular form, classifying them by the type of instrument used for their assessment and the potential impact on the quality of the SR.

### Recording the data

In order to extract the information, an outline was developed. The framework incorporates 11 categories to evaluate the full review articles included (Table 2). In order to guarantee that the outline is applied reliably by the two reviewers, it will be pilot tested by two members of the team on a subsample of the included studies. Following the data extraction outline, the same two researchers independently will record the data from each involved review study. Finally, if necessary, differences in extracted information will be discussed between the two reviewers until an agreement or by the mediation of a third reviewer of the team.

**Table 2:** Data extraction outline

Main category	Description
Title	
Authors	
Year of publication	
Objective	Define the specified objectives of the review.
Description of the population	Define if the review focuses on interventions targeting specifically boys or girls. The range of age, ethnic and socioeconomic background covered by the review.
Type of studies included in the review	Specificity the specific studies included in the systematic review.
Type of interventions included in the review	Describe the type of interventions on which the review emphases.
Type of intervention settings included in the review	Describe the settings of the interventions: e.g., community-base, family-based or school-based.
Reported outcomes	Define the main intervention outcomes described in the review (e.g., BMI, BMI Z-score, behavioral change, blood preasure)
Effectiviness	Describe the main results reported in the review
Country/region	Eg: North America, Asia, Europe, Latin America

## Discussion

Since the OSRs methodology’s purpose is only to harmonize the evidence, ethical consent is not necessary for the present protocol. In terms of diffusion, a paper will be submitted for publication in a scientific journal to describe the main results obtained through the OSRs. The products of the OSRs will offer a widespread overview of effective interventions to prevent or treat overweight or obesity in children and emphasize areas where data is debatable or absent. This study will have some strengths; first, the exhausted search will be conducted in several databases and citation searching. Second, an assessment of the quality of the review with a validated tool will be conducted. There might be limitations; for example, the study will be specific for interventions in scholarly children between 6 and 12 years, those studies that include a population from 0 to 18 years old will be missing. It will also provide health professionals and policymakers with crucial evidence for designing, financing, and delivering evidence-based interventions.

## Data Availability

Data sharing is not applicable to this article as no datasets were generated or analyzed during the current study.

## Abbreviations

OSR: Overview of systematic reviews
SRs: Systematic reviews
BMI *Z*-score: Body mass index *Z*-score
